# Control of IFN-I responses by the aminopeptidase IRAP in neonatal C57BL/6 alveolar macrophages during RSV infection

**DOI:** 10.1038/s41385-021-00402-w

**Published:** 2021-04-12

**Authors:** Carole Drajac, Daphné Laubreton, Quentin Marquant, Claire Chottin, Cécile Ferret, Edwige Bouguyon, Isabelle Schwartz-Cornil, Loredana Saveanu, Sabine Riffault, Delphyne Descamps

**Affiliations:** 1 INRAE, UVSQ, VIM, Université Paris-Saclay, Jouy-en-Josas, France; 2 Institut National de la Santé et de la Recherche Médicale, Unité UMR 1149, Centre de Recherche sur l’Inflammation, Paris, France; 3 Faculté de Médecine Xavier Bichat, Université Paris Diderot, Paris, France

## Abstract

Respiratory Syncytial Virus (RSV) is the major cause of lower respiratory tract infection in infants, in whom, the sensing of RSV by innate immune receptors and its regulation are still poorly described. However, the severe bronchiolitis following RSV infection in neonates has been associated with a defect in type I interferons (IFN-I) production, a cytokine produced mainly by alveolar macrophages (AMs) upon RSV infection in adults. In the present study, neonatal C57BL/6 AMs mobilized very weakly the IFN-I pathway upon RSV infection in vitro and failed to restrain virus replication. However, IFN-I productions by neonatal AMs were substantially increased by the deletion of Insulin-Responsive AminoPeptidase (IRAP), a protein previously involved in the regulation of IFN-I production by dendritic cells. Moreover, neonatal IRAP^KO^ AMs showed a higher expression of IFN-stimulated genes than their wild-type C57BL/6 counterpart. Interestingly, depletion of IRAP did not affect adult AM responses. Finally, we demonstrated that newborn IRAP^KO^ mice infected with RSV had more IFN-I in their lungs and eliminated the virus more efficiently than WT neonates. Taken together, early-life susceptibility to RSV infection may be related to an original age-dependent suppressive function of IRAP on the IFN-I driven-antiviral responses in neonatal AMs.

## Introduction

Human respiratory syncytial virus (RSV) is the major cause of bronchiolitis in infants and severe infections may lead to long-term effects such as asthma occurrence.^1^ To date, no vaccine is available. Preterm birth and polymorphism in innate immunity genes are risk factors clearly related to acute RSV bronchiolitis in infants.^2,3^ Moreover, the age of first exposure to RSV constitutes a key parameter in early-life susceptibility to RSV disease.^4^ The pulmonary mucosa of neonates exhibits an evolving immune environment (cellular composition, microbiota colonization) highly different from the adult mucosa that predisposes to type 2 immune responses.^5,6^ This strong bias towards type 2 immunity is implicated in the severity of the RSV infection and in priming for subsequent development of asthma in humans.^3,7^ Nevertheless, neonatal innate immune responses and their effects on RSV disease progression remain poorly described. Therefore, a better understanding of these innate mechanisms during the early-life period will allow the development of new and more effective therapeutic strategies against RSV infection.

Neonatal mice are sensitive to RSV infection, and the immunopathological imprinting induced by RSV infection in the early-life period can been revealed experimentally after a second infection at adult age.^8^ Using this well-established neonatal mouse model, it is possible to obtain a better picture of the early-life immunity to RSV, and also its long-term effects on airway pathology.^4^ Therefore, neonatal mice exhibited a poor dendritic cell (DC) mobilization and a major defect in IFN-I production and expression of several IFN-stimulated genes (ISGs) in the lungs in response to RSV infection.^9–11^ These data are in agreement with previous observations made in nasal washes from RSV-infected children in which IFN-I production has been rarely detected.^12^ Furthemore, the intranasal instillation of recombinant IFN-α prior to neonatal RSV infection protected mice against exacerbated inflammation in airways upon reexposure at adult age.^13^ Taken together, these studies point to a strong impact of IFN-I responses on type 2 immunity-biased immunopathology observed in the neonatal mouse model of RSV infection.^11,14^

IFN-I and ISGs are critical for establishing an antiviral state in infected and neighboring cells during infection by directly blocking virus replication or by modulating immune signaling pathways.^15^ In addition, IFN-I receptor-signaling amplifies the early pro-inflammatory responses in the lungs.^16^ Several pattern recognition receptors (PRRs), including Toll-like receptors (TLRs), TLR3 and TLR7, or retinoic acid-inducible gene (RIG)-I-like receptors (RLRs) detect the pathogen-associated molecular patterns (PAMPs) of RSV and induce subsequent expression of genes encoding IFN-I and ISGs through the activation of the transcription factor interferon regulatory factor 3 (IRF-3) and IRF-7.^17^ However, the sensing of viral nucleic acid by these PRRs is still poorly described in neonates.^3,7^

IFN-I production is necessary for limiting the spread of infection and its control avoids an inappropriate immune response that could cause detrimental immunopathology.^15^ Thereby, the intracellular trafficking of PRRs constitutes a major control of their activation through acquisition of diverse chaperon molecules and interactions with cytoskeletal components.^18–21^ Hence, we recently showed that the aminopeptidase IRAP (Insulin-Responsive Aminopeptidase), a protein anchored at the membrane of many intracellular vesicles and known to control the endosomal maturation,^22^ participates in IFN-I signaling in DCs.^23^ Through its interaction with the actin cytoskeleton, IRAP regulates the endosomal trafficking of TLR ligands by slowing their transit to lysosomes, thus moderating the activation of transported innate receptors and their ability to induce cytokines and IFN-I. Moreover, this recent work highlights the importance of the actin cytoskeleton in the activation process of PRRs and its regulation. The alveolar macrophages (AMs) were previously described as the main producers of IFN-I in response to RSV infection in adult mice.^24^ Recently, we observed that the ability of AMs from BALB/c mice to produce IFN-I upon RSV infection depends on age.^25^ However, the molecular mechanisms that control IFN-I pathways are poorly known in the neonatal context and remain to be deciphered in order to elucidate their age-related effects to RSV infection.

As IRAP is expressed in mouse macrophages,^26^ we investigated in this study the ability of neonatal AMs to mobilize IFN-I pathway following RSV infection, and we determined the contribution of IRAP in this response. We showed that, unlike adult AMs, neonatal AMs from C57BL/6 mice, which are permissive to RSV infection,^24,27^ were unable to generate IFN-I responses upon viral infection. Depletion of the aminopeptidase IRAP restored the secretion of IFN-α/β and the expression of ISGs in neonatal IRAP-deficient (IRAP^KO^) AMs infected with RSV, but did not affect adult AM responses. Enhanced IFN-I production in neonatal IRAP^KO^ AMs was dependent upon viral replication. We also observed that neonatal IRAP^KO^ AMs were less permissive to RSV replication than neonatal WT AMs. As previously demonstrated in adult AMs,^24^ we revealed that RIG-I/MDA-5 activation was a major innate signaling pathway responsible for IFN-I responses in neonatal AMs. Surprisingly, the absence of IRAP did not impact IFN-I responses in neonatal AMs exposed to different TLR- or RLR-agonists. Finally, we demonstrated that newborn IRAP^KO^ mice infected with RSV had a higher IFN-I production than wild-type (WT) neonates in their lungs and controlled better virus replication. Taken together, our data suggest that IRAP acts in an age-dependent manner as a supressor of IFN-I pathway following RSV infection in AMs.

## Results

### Neonatal C57BL/6 mice are permissive to RSV infection and show inadequate IFN-I pathway induction

**Fig. 1:**
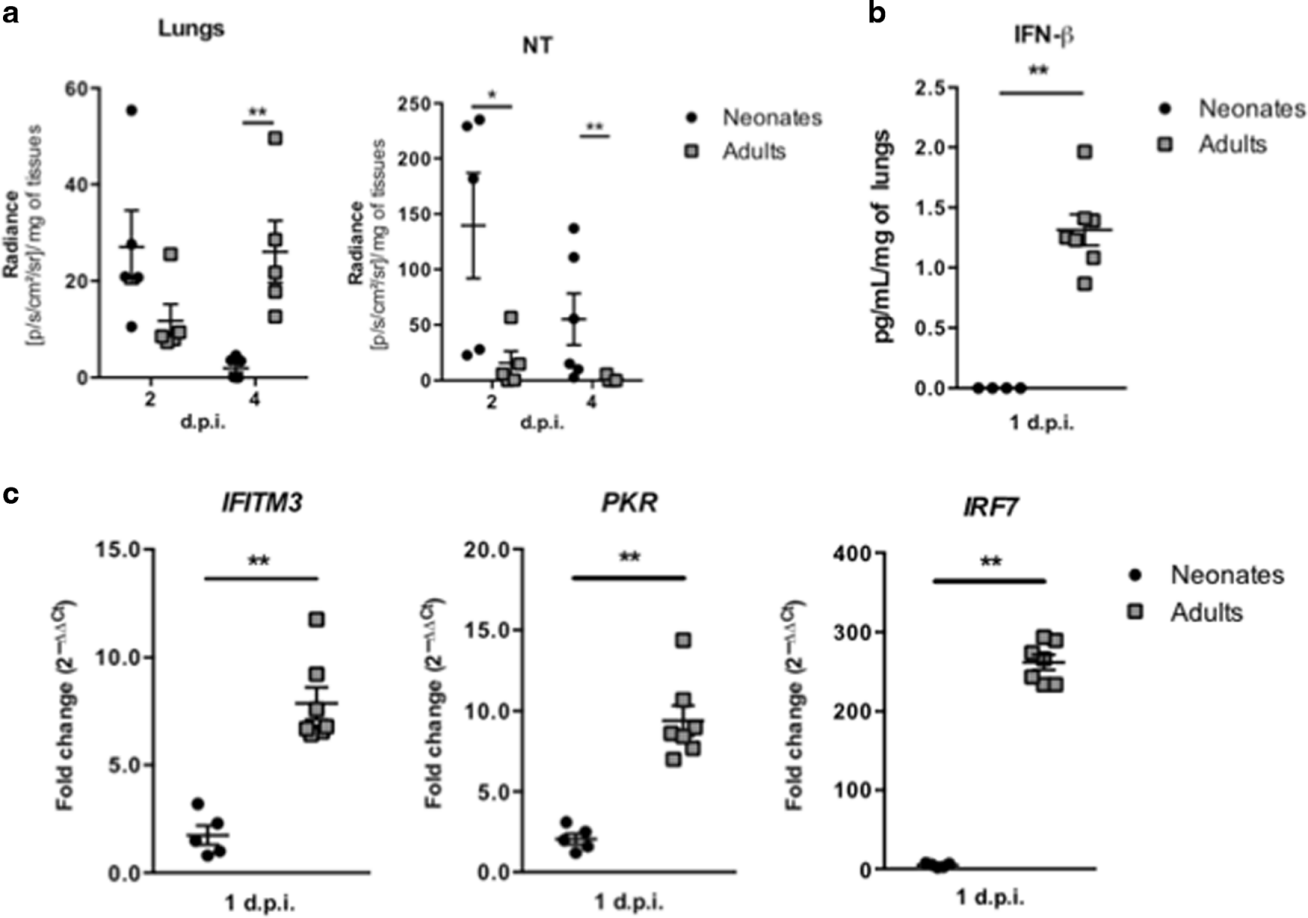
Neonatal C57BL/6 mice are permissive to RSV infection and are unable to active an efficient type I IFN pathway. Six days old neonates and 8 weeks old adult C57BL/6 mice were infected i.n. with rRSV-Luc (1.75 × 10^6^ pfu/mL, 10 µL/neonate, 50 µL/adult). **a** Quantification of luciferase activity in lung and NT homogenates (radiance in photons/s/cm^2^/sr/mg of tissues) at 2 and 4 days post-infection (d.p.i.). Results are expressed as mean of individual ± SEM (one representative experiment of two with *n* = 4–6 mice/group, *P < 0.05; **P < 0.01). **b** IFN-β production was measured by luminex assays in lung homogenates at 1 d.p.i. **c** The expressions of different ISGs and innate sensing receptors were determined by qRT-PCR on lung homogenates at 1 d.p.i. The level of expression was calculated by the formula 2^−ΔCt^ with ΔCT_RSV_ = Ct_gene_ − Ct_House keeping-genes_. Results are expressed in fold-change determined with the formula 2^−ΔΔCt^ with ΔΔCT = ΔCT_RSV_/ΔCT_mock_. Data are from two experiments. Means ± SEM are represented. **b**, **c** Results are expressed as mean of individual ± SEM (one representative experiment of three with *n* = 3–7 mice/group, *P < 0.05; **P < 0.01).

To determine the contribution of IRAP in anti-RSV responses, we used mice genetically deficient for IRAP (IRAP^KO^) produced in a C57BL/6 genetic background. Previous studies have shown that susceptibility to RSV infection depends on the genetic background of the mouse.^28^ Adult C57BL6 mice are known to be less permissive than BALB/c mice to viral infection.^29^ Recently, we demonstrated an age-dependent kinetic of RSV replication in BALB/c mice.^25^ We therefore established the kinetic of viral replication by bioluminescence in 6 days old C57BL/6 neonates infected with RSV-Luciferase (RSV-Luc).^30^ Quantification of luciferase activity in the lungs and nasal turbinates (NT) was performed in tissue homogenates at 2 and 4 d.p.i., and not in living mice due to bioluminescence absorption by the pigmentation of newborn skin (Supplementary Fig. 1). Similar to adult BALB/c mice, adult C57BL/6 mice had their maximum viral replication detected at 4 d.p.i. in lung and NT homogenates (Fig. 1a) and were able to produce IFN-β (Fig. 1b) and to mobilize IFN-I pathway in the lungs at 1 d.p.i., as illustrated by the induction of ISG gene expression (Fig. 1c). In contrast, RSV replication in neonatal C57BL/6 mice was detected at its highest level at 2 d.p.i. in the respiratory tissues (Fig. 1a). Neonatal BALB/c mice present a major defect in mobilizing the IFN-I pathway following RSV infection.^9,13,25^ The same defect was observed in RSV-infected neonatal C57BL/6 mice, as illustrated by the absence of IFN-β production (Fig. 1b) and the limited induction of ISG gene expression as compared to adult C57BL/6 mice (Fig. 1c). Taken together, these data suggest that neonatal C57BL/6 mice are rapidly permissive to RSV replication and also present a defect in mobilizing the IFN-I pathway following RSV infection.

### Neonatal primary alveolar macrophages from IRAP^KO^ mice mobilize IFN-I pathway upon RSV infection in vitro

**Fig. 2:**
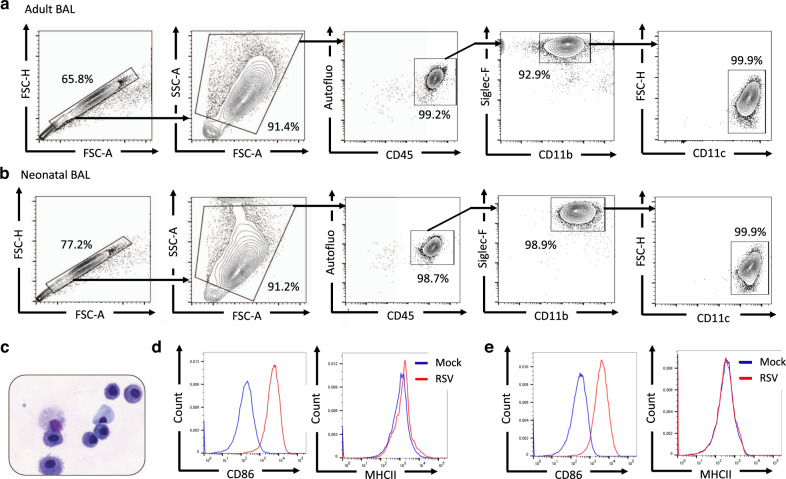
Adult or neonatal AMs collected from BAL of neonatal C57BL6 mice are activated upon RSV infection. Cells were collected from BAL of adult (**a**, **d**) or 6 days old (**b–e**) C57BL/6 mice and analyzed for phenotype by flow cytometry. Representative contour plots of adult AMs (**a**) or 6 days old mice (**b**) are shown. **c** Representative cytospin picture of BAL collected from 6 days old mice and stained with May-Grunwald Giemsa coloration. **d**, **e** AMs collected from BAL of adult animals (**d**) or 6 days old C57BL/6 mice (**e**) were plated for 24 h then infected for 24 h with RSV (MOI = 5) or Mock control. Expression level (MFI level) of CD86 or MHCII activation markers was analyzed at the cell surface of neonatal AMs by flow cytometry.

In infected adult mice, IFN-I are mainly and rapidly produced by AMs that are permissive to RSV infection although the viral replication is abortive.^24,27,31^ Recently, we demonstrated that neonatal primary AMs collected from BALB/c mice display lower antiviral and inflammatory responses to RSV infection in vitro than adult AMs.^25^ First, we characterized the purity and the phenotype of cells collected from bronchoalveolar lavages (BAL) of adult or neonatal C57BL/6 WT mice by FACS analysis. We showed that cells isolated from adult (Fig. 2a) and neonatal BAL (Fig. 2b) were similar and formed an homogeneous population, with the typical phenotype autofluorescence^+^ Siglec-F^+^ CD11b^+^ CD11c^+^ and the morphologic characteristics of AMs (Fig. 2c). Exposure of adult AMs (Fig. 2d) or neonatal AMs (Fig. 2e) to RSV enhanced the expression of co-stimulatory molecules illustrated by the increase of CD86 median of fluorescence intensity (MFI) at the cell surface after 24 h of infection, thus demonstrating their ability to respond to viral infection. Adult AMs presented more MHCII molecules on their surface than neonatal cells (Fig. 2d, e, peak MFI of 1131 for adult AMs and of 178 for neonatal AMs). However, whatever the age of AMs, this expression was not increased upon RSV infection under our experimental conditions (Fig. 2d, e).

**Fig. 3:**
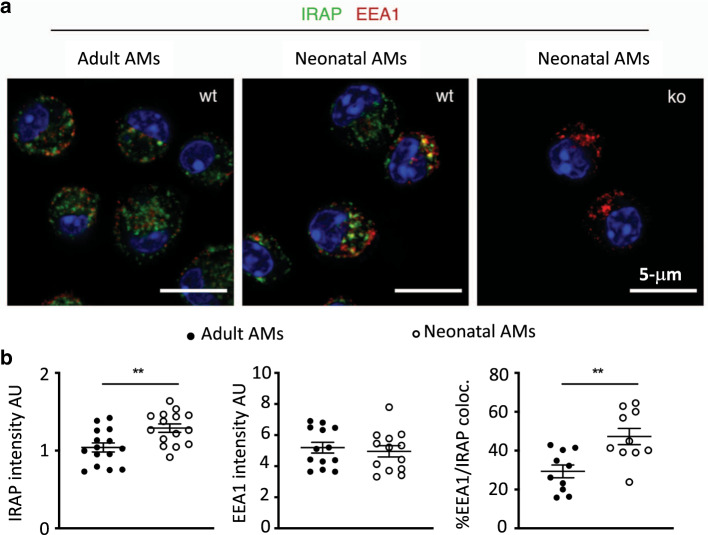
Intracellular localization of IRAP in adult or neonatal AMs. AMs collected from BAL of adult C57BL/6 mice or 6 days old C57BL/6 mice or 6 days old IRAP-deficient mice were plated for 24 h. **a** Cells were fixed and stained for IRAP (Green), early endosome antigen 1 (EEA1), an early endosomal marker (red), and nuclear DAPI labeling (blue). Bars represent 5 μm scale. **b** The quantification of IRAP and early endosomal marker (EEA1) and their co-localization were performed (*n* = 15 cells for each labeling). Image treatment and analysis were performed with ImageJ software. Means ± SEM are represented. **P < 0.01.

**Fig. 4:**
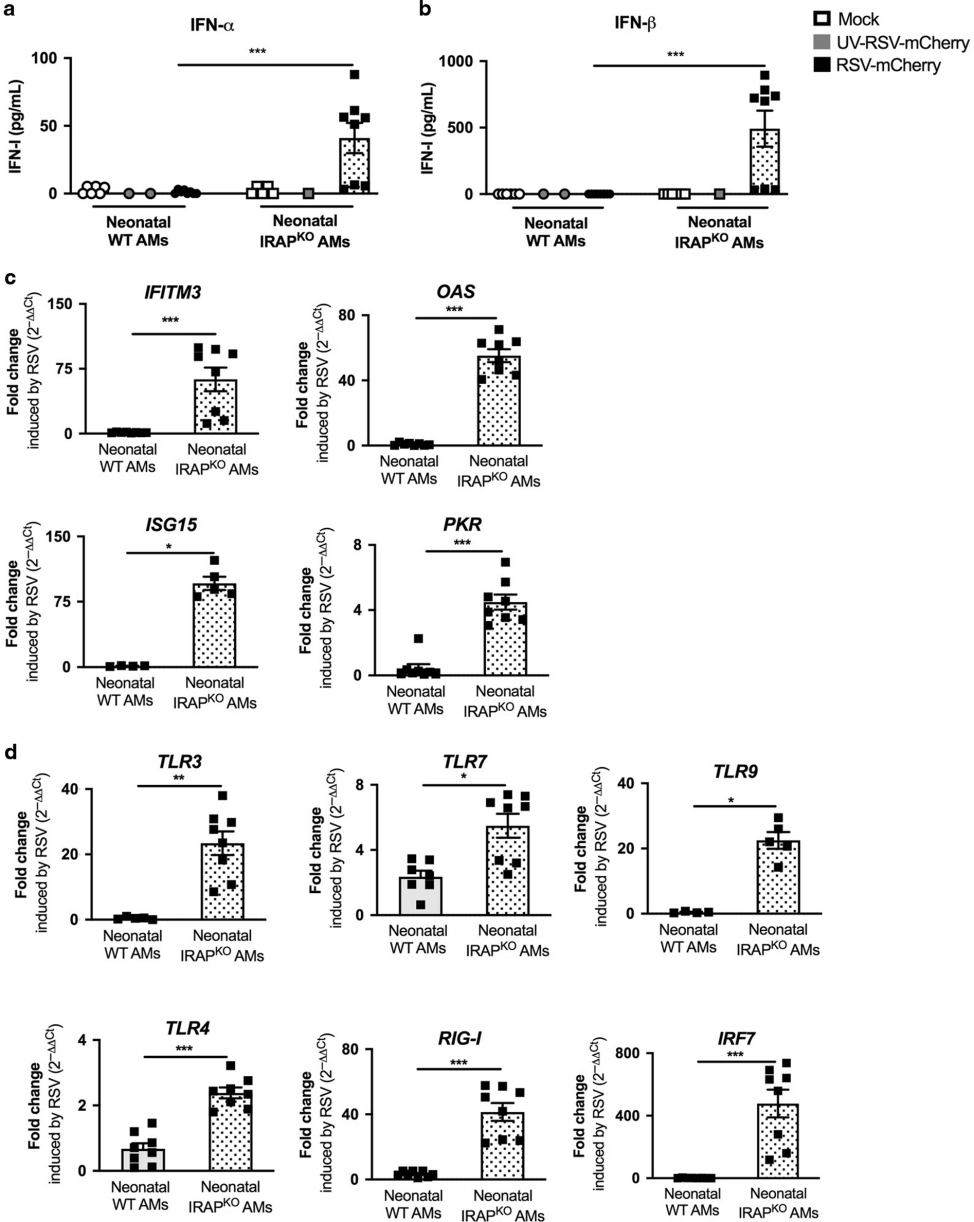
Deletion of IRAP restores type I IFN pathway in neonatal AMs following in vitro RSV infection. AMs isolated from BAL of neonatal C57BL/6 wild-type (WT) or IRAP-deficient (IRAP^KO^) mice were exposed to Hep2-supernatant (Mock, white symbol) or rRSV-mCherry (RSV-mCherry, black symbol) or UV-inactivated rRSV-mCherry (UV-RSV-mCherry, gray symbol) at MOI of 5 for 2 h. The productions of IFN-α (**a**) and IFN-β (**b**) were measured 24 h post-infection in supernatants using ProcartaxPlex immunoassay. The expressions of different ISGs (**c**) and innate sensing receptors (**d**) were determined by qRT-PCR on cell lysates 24 h post-infection. The level of expression was calculated by the formula 2^−ΔCt^ with ΔCT_RSV_ = Ct_gene_ − Ct_GADPH_. Results are expressed in fold-change determined with the formula 2^−ΔΔCt^ with ΔΔCT = ΔCT_RSV_/ΔCT_mock_. Data are from two experiments. Means ± SEM are represented. *P < 0.05; **P < 0.01; ***P < 0.001.

Since the amplitude of IFN-I production is regulated by IRAP in DCs upon TLR stimulation,^23^ we sought to determine whether the deletion of IRAP could affect the IFN-I pathway in AMs upon RSV infection. To investigate the intracellular localization and the expression level of IRAP, we stained adult or neonatal AMs with a marker of early endosome (Early Endosomal Antigen 1, EEA1) and found that IRAP colocalizes with EEA1 at steady state in both adult and neonatal AMs (Fig. 3a, b, mean % co-localization of 47.3 in neonatal AMs and 29.3% in adult AMs), as previously described in DCs.^22^ We also showed that neonatal AMs have a higher intensity of intracellular IRAP expression than adult cells (Fig. 3b). To address the ability of neonatal AMs collected from the C57BL/6 genetic background and the role of IRAP to mobilize IFN-I pathway in response to RSV infection, we compared the production of IFN-I and the induction of ISGs, 24 h after exposure to recombinant RSV-mCherry in neonatal C57BL/6 WT (WT AMs) or IRAP-deficient AMs (IRAP^KO^ AMs). IFN-α (Fig. 4a) and IFN-β (Fig. 4b) productions by neonatal WT AMs in response to RSV infection were undectable in culture supernatants. IFN-I induces the expression of several ISGs that are known to interfere with viral replication and viral spread, and thus contributes to the early control of infection. Some PRRs and associated-signaling molecules that participate to the IFN-I pathway like TLR3, TLR7, RIG-I, or IRF7, are also upregulated in order to amplify IFN-I response.^15,32^ qRT-PCR analyses showed that upon RSV infection, the calculated ratio of *mRNA* levels between RSV and mock conditions (fold change), for genes belonging to ISG family (*IFITM3*, *OAS*, *ISG15*, and *PKR*, Fig. 4c) or involved upstream in IFN-I pathways (*TLR3*, *TLR9*, *RIG-I*, or *IRF7*, Fig. 4d) were at very low levels in neonatal WT AMs. However, we observed that the expression levels of TLR4 and TLR7 were increased in neonatal WT AMs in response to RSV. These data demonstrated that neonatal WT AMs did not mobilize efficiently IFN-I pathway upon RSV infection.

IFN-α and IFN-β productions upon infection with RSV were stronger in neonatal IRAP^KO^ AMs (41.0 ± 31.7 pg/mL and 492.7 ± 384.1 pg/mL, respectively, Fig. 4a, b) than in neonatal WT AMs (1.08 ± 1.31 pg/mL and undectable, respectively, Fig. 4a, b). This response was abrogated by UV-inactivation of RSV (UV-RSV-mCherry), suggesting that the enhanced IFN-I production is dependent on viral replication in AMs (Fig. 4a, b). To further investigate the role of IRAP in IFN-I signaling, we showed that the level of expression for all ISG and PRR upstream in IFN-I pathways induced by RSV was much stronger in neonatal IRAP^KO^ AMs than in neonatal WT AMs (Fig. 4c, d). Thus, neonatal IRAP^KO^ AMs were fully competent to mobilize IFN-I pathway upon RSV infection in vitro.

### Adult primary AMs from WT or IRAP^KO^ mice exposed to RSV in vitro produce IFN-I

**Fig. 5:**
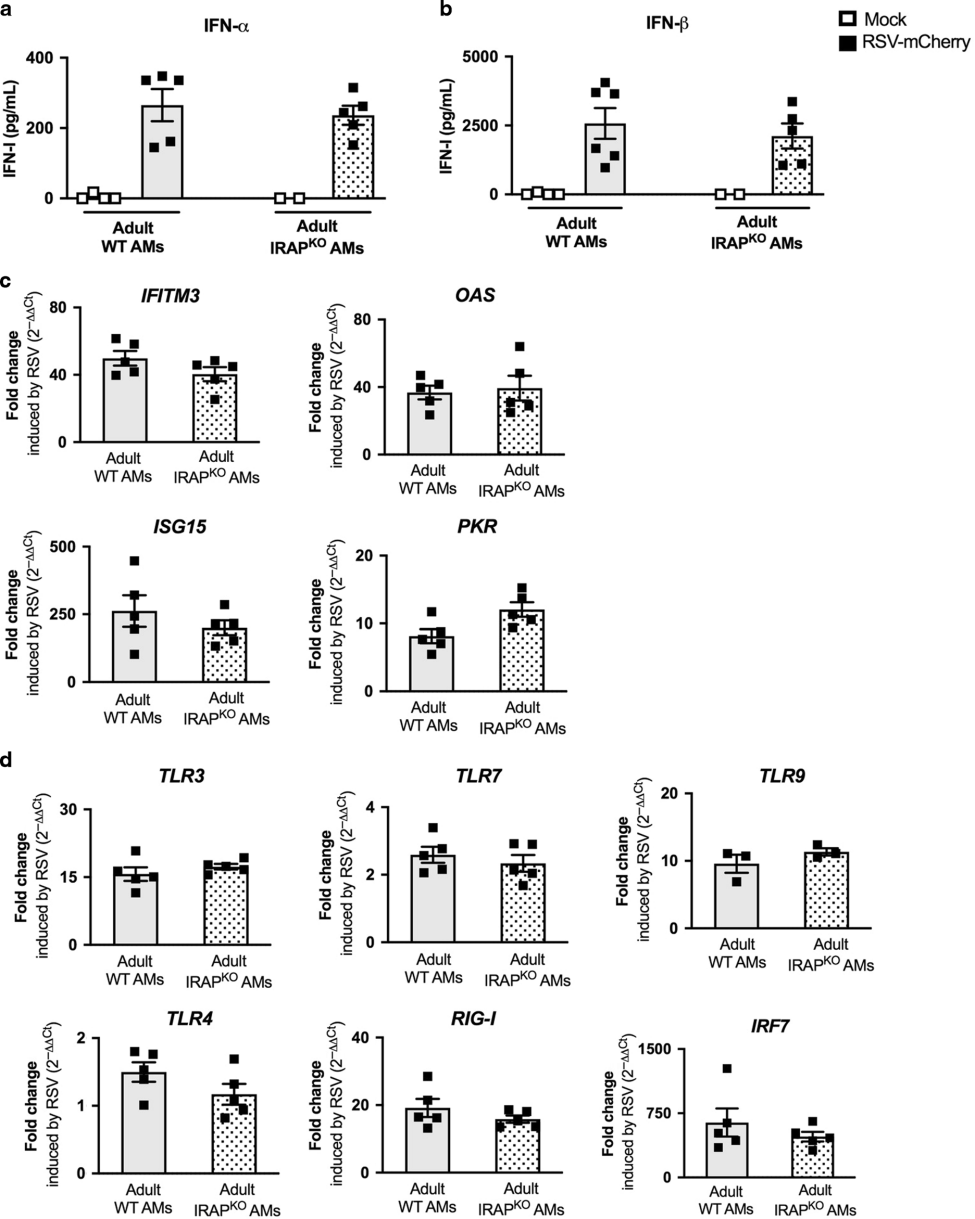
Deletion of IRAP in adult AMs does not affect the type I IFN response to in vitro RSV infection. Adult AMs from WT C57BL/6 or IRAP-deficient (IRAP^KO^) mice were exposed to Hep2-supernatant (Mock, white symbol) or rRSV-mCherry (RSV-mCherry, black symbol) at MOI of 5 for 2 h. The productions of IFN-α (**a**) and IFN-β (**b**) were measured 24 h post-infection in supernatants using ProcartaxPlex immunoassay. The expressions of different ISGs (**c**) and innate sensing receptors (**d**) were determined by qRT-PCR on cell lysates 24 h post-infection. The level of expression was calculated by the formula 2^−ΔCt^ with ΔCT_RSV_ = Ct_gene_ − Ct_GADPH_. Results are expressed in fold-change determined with the formula 2^−ΔΔCt^ with ΔΔCT = ΔCT_RSV_/ΔCT_mock_. Data are from two experiments. Means ± SEM are represented.

We also analyzed the role of IRAP in RSV-induced IFN-I responses of adult AMs. As previously described,^24^ AMs collected from adult WT C57BL/6 mice secreted large amounts of IFN-α (Fig. 5a) and IFN-β (Fig. 5b) in response to RSV. Morever, a strong upregulation of ISG and PRR expression was induced following RSV infection, as illustrated by RSV-induced fold change (Fig. 5c, d). However, the absence of IRAP in adult AMs had no effect on IFN-I production (Fig. 5a, b) and ISG (Fig. 5c) or PRR inductions (Fig. 5d) following RSV infection. Collectively, these results demonstrated that IRAP controlled the activation of IFN-I pathway in neonatal C57BL/6 AMs exposed to RSV but not in adult AMs, suggesting an age-dependent involvement of IRAP in IFN-I responses.

### An effective IFN-I signaling in response to RSV infection is necessary to restrain virus replication in AMs

**Fig. 6:**
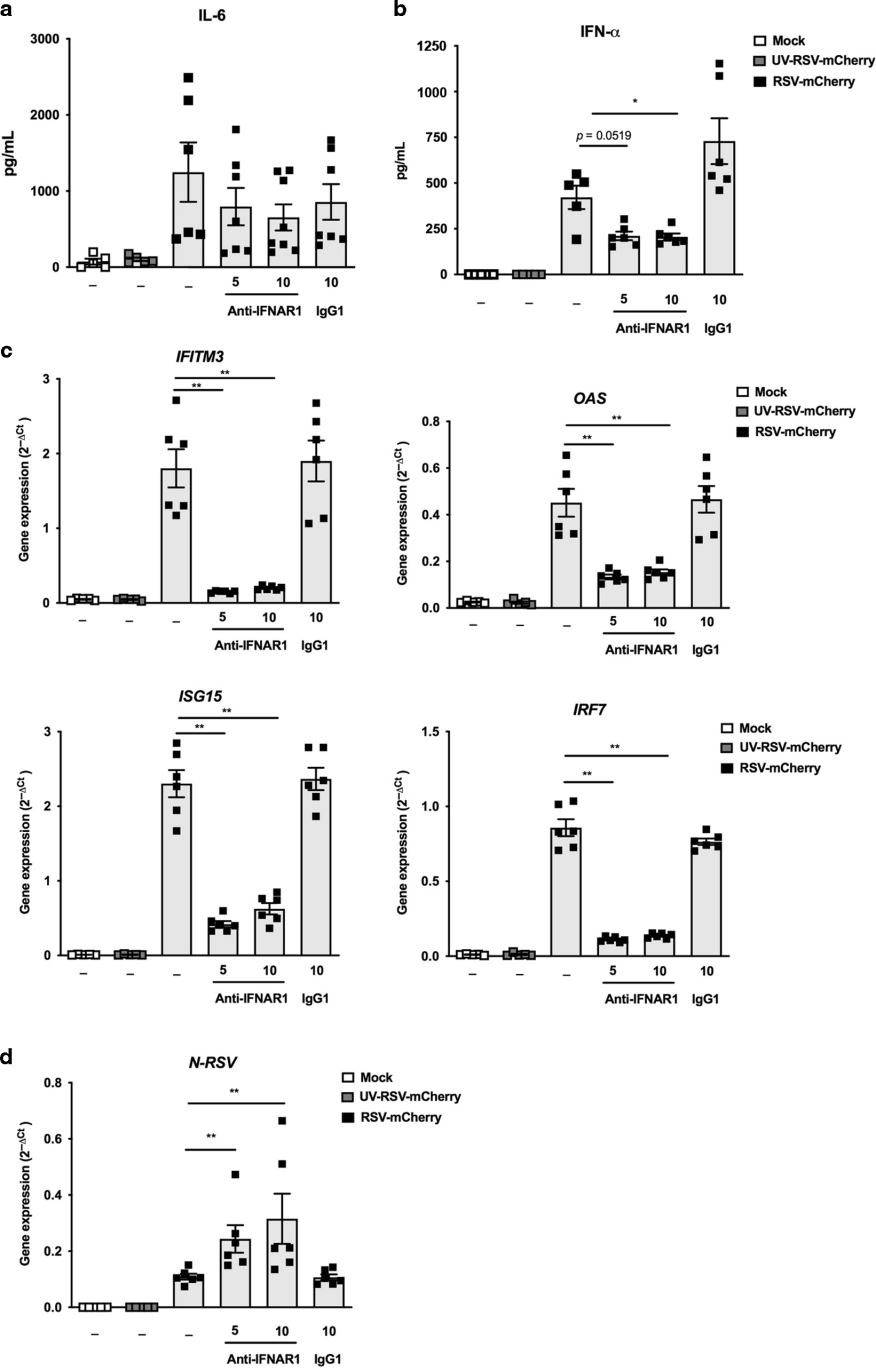
RSV replication in AMs is retrained by induction of type I IFN signaling. **a–d** Adult AMs collected from BAL of WT C57BL/6 mice were exposed to Hep2-supernatant (Mock, white symbol) or rRSV-mCherry (RSV-mCherry, black symbol) or UV-inactivated rRSV-mCherry (UV-RSV-mCherry, gray symbol) at MOI of 5 for 2 h in the presence or not of anti-IFNα/β receptor (IFNAR) antibody (5 or 10 μg/mL). The productions of IL-6 (**a**) or IFN-α (**b**) were measured 24 h post-infection in supernatants using ELISA or ProcartaxPlex immunoassay, respectively. **c** The expressions of different ISGs were determined by qRT-PCR on cell lysates 24 h post-infection. **d** RSV replication was evaluated by qRT-PCR of *N-RSV* gene expression on cell lysates 24 h post-infection. The level of expression was calculated by the formula 2^−ΔCt^ with ΔCT = Ct_RSV_ − Ct_GADPH_. **d** Data are from two experiments. Means ± SEM are represented. *P < 0.05; **P < 0.01; ***P < 0.001.

The restrained viral production during the first days of infection in adult mice is dependent on the induction of IFN-I pathway.^9,24^ Thus, we evaluated if the inhibition of interferon-α/β receptor (IFNAR) signaling could impact the ability of adult AMs to restrain RSV infection. To address this issue, adult WT AMs were infected with recombinant RSV-mCherry for 24 h, with or without IFNAR blocking antibody. In these experimental settings, the secretion of inflammatory cytokine, interleukine-6 (IL-6) of RSV-infected adult AMs was not affected (Fig. 6a). However, IFN-α production (Fig. 6b) and ISG expressions (Fig. 6c) of RSV-infected adult AMs were decreased by the presence of IFNAR blocking antibody while RSV replication, illustrated by the level of expression of the viral *N-RSV RNA* was increased (Fig. 6d). The level of expression of mCherry protein could not be determined because the signals were too weak for robust measurements (not shown). Thus, the activation of the IFN-I pathway in AMs exposed to RSV had a major impact on the ability of adult cells to restrain viral production.

**Fig. 7:**
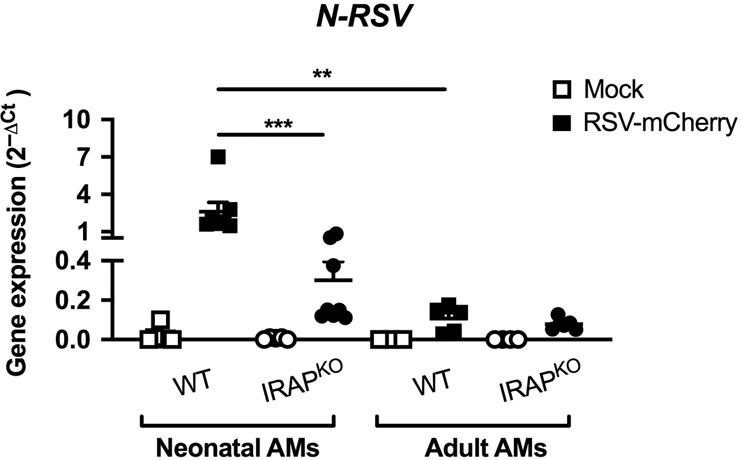
Neonatal IRAP^KO^ AMs restrain more efficiently the RSV replication than neonatal WT AMs. Neonatal or adult AMs collected from BAL of wild-type (WT) C57BL/6 mice or IRAP-deficient (IRAP^KO^) mice were exposed to rRSV-mCherry (RSV-mCherry, black symbol) at MOI of 5 or Hep2-supernatant (Mock, white symbol) for 2 h. *N-RSV RNA* expression was quantified by qRT-PCR on cell lysates 24 h post-infection. Results were calculated by the formula 2^−ΔCt^ with ΔCT = Ct_RSV_ − Ct_GADPH_. Data are from two experiments. Means ± SEM are represented. **P < 0.01; ***P < 0.001.

We showed that, in contrast with antiviral adult AM responses, AMs from neonatal WT C57BL/6 mice failed to mobilize IFN-I pathway upon RSV infection in vitro (Fig. 4). As expected, significantly more *N-RSV RNA* was detected in neonatal WT AMs than in adult WT AMs (Fig. 7), demonstrating that neonatal C57BL/6 AMs were more permissive to viral replication than adult AMs. *N-RSV RNA* in neonatal IRAP^KO^ AMs was detected at significantly lower levels than in neonatal WT AMs (8.6-times, Fig. 7). Altogether, neonatal IRAP^KO^ AMs were less permissive to the viral replication than neonatal WT AMs in agreement with their potent IFN-I responses.

### IFN-I production trigerred by TLR- or RIG-I/MDA-5-ligands in neonatal AMs is independent of IRAP

**Fig. 8:**
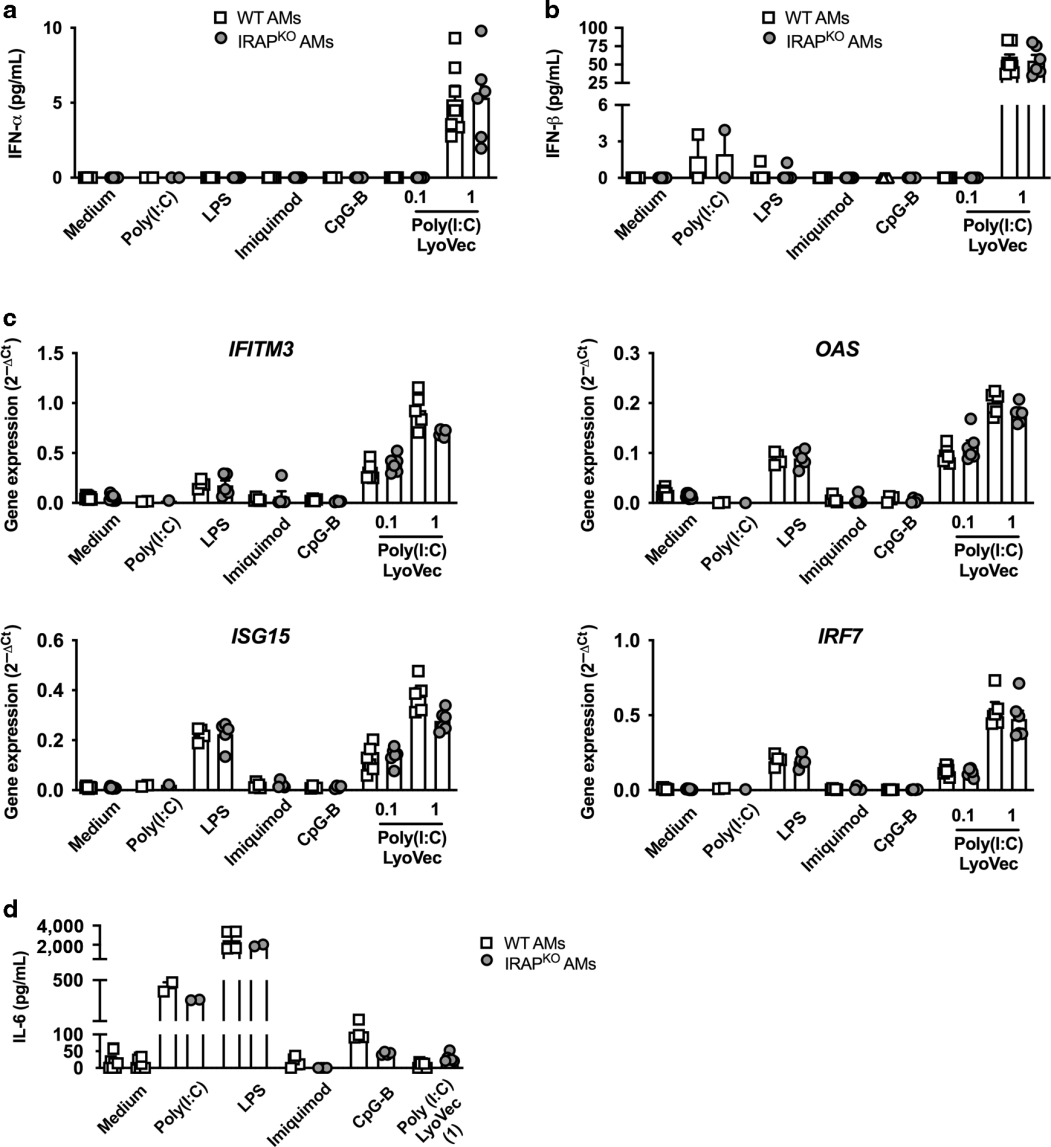
Type I IFN and inflammatory responses upon specific innate sensing receptor stimulation do not depend upon IRAP expression in neonatal AMs. Neonatal AMs from wild-type (WT, white square) or IRAP-deficient (IRAP^KO^, gray circle) mice were stimulated with different PAMPs: Poly(I:C) (100 μg/mL), LPS (10 µg/mL), Imiquimod (100 μg/mL), CpG-B (100 μg/mL), or Poly(I:C)-LyoVec (0.1 or 1 μg/mL). The productions of IFN-α (**a**) and IFN-β (**b**) were measured 24 h post-infection in supernatants using ProcartaxPlex immunoassay. **c** The expression of different ISGs was determined by qRT-PCR performed on cell lysates 24 h post-infection. The level of expression was calculated by the formula 2^−ΔCt^ with ΔCT = Ct_gene_ − Ct_GADPH_. Data are from four experiments. Means ± SEM are represented. **d** The production of IL-6 was measured 24 h post-stimulation in supernatants using ELISA. Data are from four experiments. Means ± SEM are represented.

We next explored the pathways leading to IFN-I induction in neonatal AMs and their sensitivity to IRAP control. Neonatal WT and IRAP^KO^ AMs were stimulated with specific TLR ligands or RLR-agonist, such as the synthetic RNA duplex Poly(I:C), lipopolysaccharide (LPS), imiquimod, CpG-B or the transfected Poly(I:C) (Poly(I:C)-LyoVec) which are specific TLR ligands or RLR-agonist for TLR3, TLR4, TLR7, TLR9 and RIG-I/MDA-5, respectively. The IFN-I and inflammatory responses were analysed after 24 h of stimulation. Our data revealed that only stimulation with Poly(I:C)-LyoVec (1 μg/mL) induced a significant secretion of IFN-α (Fig. 8a) and IFN-β (Fig. 8b) by neonatal AMs, with no differences between WT and IRAP^KO^ neonatal AMs. qRT-PCR analyses showed that the *mRNA* levels of ISGs (*IFITM3*, *OAS*, *ISG15*, and *IRF7*) were equivalently upregulated in both neonatal WT and IRAP^KO^ AMs upon stimulation with LPS and Poly(I:C)-LyoVec (0.1 and 1 μg/mL) (Fig. 8c). Nevertheless, Poly(I:C), LPS and CpG-B-stimulated neonatal AMs (WT and IRAP^KO^) produced interleukine-6 (IL-6), demonstrating their ability to respond to TLR3-, TLR4-, and TLR9-stimulation (Fig. 8d). In these experimental settings, Imiquimod/TLR7 signaling did neither induce IFN-I pathways nor IL-6 in neonatal AMs, whatever the WT or IRAP^KO^ context. The same observation was made with adult WT and IRAP^KO^ AMs in which RIG-I/MDA-5 stimulation induced similar IFN-I and ISG levels (Supplementary Fig. 2), levels that were even similar with those of the neonatal AMs (Fig. 8). In conclusion, we showed that neonatal AMs, like adult AMs, can produce IFN-I upon RIG-I/MDA-5 but not TLR stimulation, in an IRAP-independent manner. Therefore, the control of IFN-I responses by IRAP in neonatal AMs appeared selective to RSV signaling, within the range of stimuli tested in this study.

### Enhanced IFN-I production and better control of virus replication in the lungs of newborn IRAP-deficient mice

**Fig. 9:**
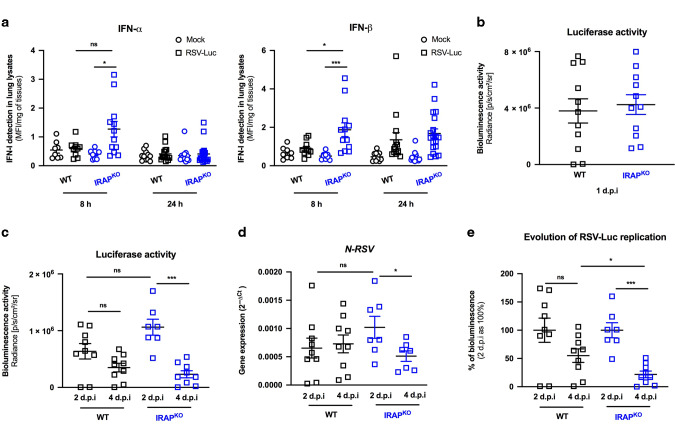
IRAP-deficient newborn mice display an enhanced production of type I IFNs in the lungs and better control of RSV replication. Wild-type (WT) or IRAP-deficient (IRAP^KO^) neonatal mice were infected with Hep2-supernatant (Mock) or rRSV-Luc (RSV-Luc). **a** The productions of IFN-α and IFN-β were measured post-infection in lung lysates using ProcartaxPlex immunoassay. Results are expressed in mean fluorescent intensity (MFI) per mg of lung lysates at 8 or 24 h post-infection. **b**, **c** Luciferase activity associated to viral replication was measured in lung lysates (left lung lobe) by quantification of photon emission (radiance in photon/sec/cm^2^/sr). **d** N-RSV gene expression was measured in the lung lysates by qRT-PCR and calculated by the formula 2^−ΔCt^ with ΔCT = Ct_N-RSV_ − Ct_GADPH_. **e** Reduction of RSV replication between 2 d.p.i and 4 d.p.i. Each individual bioluminescence signal obtained at 2 d.p.i and 4 d.p.i was normalized to the mean bioluminescence value of each group at 2 d.p.i considered as 100%. **a** Data are mean ± SEM from three independent experiments with *n* = 8–14 mice per group and timepoints. **b–e** Data are mean ± SEM from two independent experiments with *n* = 7–11 mice per group and timepoints. *P < 0.05; **P < 0.01; ***P < 0.001.

Our ex vivo data demonstrates that IRAP controlled IFN-I responses in neonatal AMs exposed to RSV. We next addressed whether neonatal mice deficient in IRAP (IRAP^KO^ 6 days old mice) displayed enhanced production of IFN-I following infection with RSV-Luc. As previously described,^9,13^ the production of IFN-I was barely detectable in the lungs of RSV-infected WT neonates even at very early timepoints (Figs. 1b,  9a). In contrast, RSV infection of IRAP^KO^ neonatal mice induced two times more IFN-α and IFN-β respectively in their lungs, 8 h after infection, relatively to WT mice (Fig. 9a). No differences were observed 24 h after infection. We next measured the viral replication in the lungs of infected neonatal WT and IRAP^KO^ mice to investigate whether they differ in their capacity to control RSV replication. At 24 h after RSV exposure, the luciferase activity in the lungs were similar (Fig. 9b), demonstrating similar ability of the virus to replicate in newborn WT and IRAP^KO^ mice at the onset of infection. From 2 d.p.i, the luciferase activity (Fig. 9c) and the level of N-RSV mRNA (Fig. 9d) in the lungs decreased in both WT and IRAP^KO^ neonates. Interestingly, the reduction of RSV replication between 2 d.p.i and 4 d.p.i was more pronounced and only significant in the lungs of IRAP^KO^ neonates (Fig. 9c, d). Individual bioluminescence signals obtained at 2 and 4 d.p.i were then normalized by the mean bioluminescence value of each group at 2 d.p.i considered as 100%. This analysis showed that more than 78% of RSV-Luc-dependent bioluminescence was eliminated from the lungs of IRAP^KO^ neonates between 2 d.p.i and 4 d.p.i while the decrease of luciferase activity was only 45% in WT mice (Fig. 9e). Thus, RSV elimination at 4 d.p.i was significantly greater in the lungs of IRAP^KO^ neonates. Taken together, these results demonstrated that the lack of IRAP enhanced the early IFN-I production in the lungs of neonatal mice and altered the kinetic of RSV replication.

## Discussion

IFN-α and IFN-β are critical for controlling viral pathogenesis by inducing the expression of several ISGs that provide a robust first line of defense against viruses. Moreover, IFN-I pathway also plays an important role in shaping the inflammatory and adaptive immune responses.^15,16,32^ The early-life susceptibility to RSV disease has been associated with the inability of the young (infants and neonatal BALB/c mice) to mobilize the IFN-I pathway in the lungs of upon infection.^9,12,13,25^ Nevertheless, the molecular mechanisms that control IFN-I pathway are poorly described in the context of neonates. In this study, we identified IRAP as a negative regulator of RSV replication-mediated IFN-I pathway in AMs and in the lungs during the neonatal period.

In contrast to what has been described in adult cells,^24^ RSV infection failed to induce a robust production of IFN-α/β and the upregulation of ISG transcripts in neonatal AMs from C57BL/6 mice. We recently described the capacity of neonatal BALB/c AMs to mount in vitro IFN-I and inflammatory responses following RSV infection, albeit at a lower level than adult AMs.^25^ It is known that the resistance and the immune response to RSV infection is determined by the genetic background of mouse.^28,29^ In the same way, bone marrow-derived macrophages (BMDMs) generated from C57BL/6 or BALB/c were previously described to produce distinct panel of cytokines upon TLR2 or TLR4 stimulation depending on both IFN-I and IL-10.^33^ Host genetic variants of viral infection may impact at several levels on the biological functions or mechanisms, such as i.e., virus receptor/sensor, receptor-modifying enzyme, and signaling molecules.^34^ The complete dissection of the mechanisms underlying these contrasted phenotypes is difficult due to the IFN-I pathway complexity and to the immune cell evolution with the age of the individuals. In particular, RSV susceptibility observed in infants is related to immune characteristics of the lungs in the neonatal period.^7,35^ Nevertheless, our present investigation extends to another genetic background, C57BL/6, the observation made in BALB/c mice of a weak neonatal mobilization of the IFN-I pathway upon RSV infection.^9,13,25^

This inability of WT AMs from C57BL/6 neonates to make IFN-I could be explained by age-specific requirements for activation of PRRs and/or for the acquisition of signaling components, such as the nuclear translocation of IRF-3 or IRF-7, which is impaired in neonates.^36,37^ RIG-I is recognized as being the main PRR responsible for IFN-I responses in adult WT AMs upon RSV infection.^24^ Here, we showed that IFN-α/β were secreted by WT AMs only after stimulation with a synthetic RIG-I/MDA-5-agonist, and with the same amplitude of secretion in both neonatal and adult WT AMs. Thus, neonatal WT AMs had a competent RIG-I/MDA-5-mediated IFN-I pathway, but RSV was not able to trigger IFN-I synthesis in these cells. RSV has evolved two nonstructural viral proteins, NS1 and NS2, to degrade or to sequester multiple proteins involved in recognition of PAMPs and/or innate immune signaling pathway that affect both induction and effector functions of IFN-I and type III interferons (IFN-III),^38^^.^^39^ RSV infection of human monocyte-derived DCs induces the production of a subset of IFN-I and IFN-III molecules.^40^ However, the ability of AMs (neonates or adults) to produce IFN-III following RSV infection remains to be investigated. NS1 and NS2 inhibit IFN-I production in RSV-infected epithelial cell lines by interfering with the activation of IRF-3 and its nuclear translocation, or by inhibiting RIG-I/MAVS interaction.^41,42^ Although NS1 and NS2 expression upon RSV infection reduce IFN-I production in macrophages derived from primary human peripheral blood monocytes,^43^ the role and the inhibitory effect of NS1 and NS2 have never been studied in the context of AMs infection nor during the neonatal period. Of particular interest would be the analysis of the intracellular localization of NS1 and NS2 proteins in regard to a potential co-localization with IRAP. In addition, it is possible that RSV infection induces the expression of natural inhibitors of PRR pathways, such as LGP2 (Laboratory of Genetics and Physiology 2), a member of the RLR family, which is a known cellular regulator of both RIG-I and MDA-5.^44^ To our knowledge, no study has investigated its role in RSV infection.^44^ A potential over-expression of LGP2 upon neonatal RSV infection should be considered, taking into account the study of Si-Tahar’s group, which demonstrated a decrease of IFN-I and pro-inflammatory mediators during influenza virus (H3N2) infection in mice overexpressing human LGP2.^45^

On the other hand, the intracellular trafficking of PRRs also exerts a regulatory constraint on IFN-I pathway through their mobility toward the different intracellular compartments required for PRR activation.^18–21^ Thus, the interaction of actin cytoskeleton with IRAP^+^ endosomes controls the early steps of endosomal trafficking of TLR ligands and subsequently moderates their ability to induce cytokines and IFN-I in DCs.^23^ Here, we show that neonatal IRAP^KO^ AMs made substantial amount of IFN-I and upregulated ISG transcripts leading to a significant decrease of RSV replication as compared with neonatal WT AMs. However, the viral replication was not completely blocked in neonatal IRAP^KO^ AMs. This could be explained by the lower amounts of ISG15, a protein responsible for ISGylation similar to ubiquitination, and of PKR, an inhibitor of cellular and viral mRNA translation, detected in neonatal IRAP^KO^ AMs than in adult AMs during RSV infection.^32^

The negative control exerted by IRAP on the IFN-I pathway has also been demonstrated in vivo in the lungs of RSV-infected newborn IRAP^KO^ mice. Although still weak, the pulmonary production of IFN-I was increased in the absence of IRAP following RSV infection. However, the viral replication in newborn IRAP^KO^ mice was not inhibited at the onset of infection. Nevertheless, we recorded a greater elimination of the virus in the lungs of newborn IRAP^KO^ mice at a later stage of infection (4 d.p.i). The discrepancy observed in newborn IRAP^KO^ mice between viral replication in lung tissues and in AMs could be explained by the difference between a productive viral cycle in epithelial cells and abortive cycle in AMs, but also by the biological complexity existing in the whole tissue (cellular crosstalk, several types of cells). Moreover, the deletion of IRAP expression can have several effects on both endocytic trafficking and immune cell functions, such as ability of DCs to cross-present antigens and prime adaptive immune responses, as well as the control of innate and adaptive immune receptor signaling and modulation of inflammatory responses.^22,23,46,47^ A mouse model with conditional IRAP gene knockout in AMs would be more relevant in determining the regulatory role of IRAP on the IFN-I pathway in vivo. In addition, further studies are required to determine the role of IRAP in pulmonary epithelial cells, the main cellular target for RSV replication, a role that may be different due to a productive viral cycle in epithelial cells, as compared to an abortive cycle in AMs.^27^ Finally, it would also be important to analyse the role of IRAP in the interstitial macrophages of the lungs which are known to have different functions than AMs.^48^

TLR3-, TLR7-, or TLR9-pathways could have been influenced by IRAP control, since IRAP affects their endosomal trafficking/maturation in DCs.^23^ Surprisingly, the deletion of IRAP in neonatal and adult AMs did not change their capacity to produce IFN-I and IL-6 upon exposure to the respective synthetic agonists of TLR3, TLR7 and TLR9, indicating that IRAP control can be cell type-specific. Moreover, the production of IFN-I measured after exposure to RIG-I agonist remained similar in neonatal IRAP^KO^ and WT AMs. These data suggested that the IRAP-dependent control of IFN-I pathway was specific to RSV replication in neonatal AMs. This control mechanism seems to require viral replication since the enhanced mobilization of IFN-I pathway observed in neonatal IRAP^KO^ AMs was not observed upon exposition to UV-irradiated RSV. Recently, newly synthetized viral RNA have been detected in inclusion bodies-associated granules (IBAGs) of human epithelial cell line infected by RSV.^49^ These data suggest that these IBAGs constitute the main sites of viral RNA synthesis and thus potentially concentrate RSV PAMPs. It will be interesting to characterize the presence of these IBAGs in AMs upon RSV infection and see whether IRAP plays a role in their induction.

A growing body of evidence supports the hypothesis that autophagy influences virus replication as well as IFN-I and cytokine responses upon RSV infection in macrophages, DCs and epithelial cells.^50–52^ Thus, Pokharel et al. showed that autophagy is necessary for IFN-β production *via* the activation of TGF-β—SMAD2/3 signaling pathway in RSV-infected BMDMs.^53^ By using transgenic mice expressing a recombinant LC3 protein coupled to the green fluorescent protein GFP, it has been shown that autophagy is particularly strong in the lungs, heart and other neonatal organs during the first hours of life.^54^ Autophagy plays also a critical role in the acquisition of age-dependent features in macrophages. Indeed, BMDMs from aged mice (>100 weeks) exhibit reduced autophagic process compared to young mice (6–8 weeks).^55^ Today the link between autophagy and IRAP remains elusive. However, since autophagy contributes to IFN-I production in RSV-infected BMDMs^53^ and because autophagy diminishes rapidly with age,^54^ we propose the hypothesis that IRAP could control IFN-I response to RSV infection in neonates *via* the regulation of autophagy. Indeed, IRAP has been shown to interact with several proteins involved in vesicle formation or in cytoskeleton remodeling such as vimentin,^56^ and different formins.^23,57^ In addition, IRAP colocalizes in DCs or in adipocytes with the Rab11 or Rab14 GTPase, proteins required in the autophagic process and for the assembly and budding of RSV at the apical surface of human epithelial cells.^22,58–61^ In order to understand how IRAP could mechanistically affect autophagy in AMs, it would be interesting to analyze the potential interaction of IRAP with proteins participating in the autophagic process such as LC3, beclin or Rab proteins.

Finally, we did not observe any modification of the IFN-I responses in adult IRAP^KO^ AMs compared to WT cells upon RSV infection. These data suggested that IRAP exert an age-dependent suppressive function on IFN-I pathway in AMs, in the particular context of the neonatal airway mucosa whose immunological characteristics are rapidly evolving.^7^ In conclusion, our study has revealed IRAP as a key element in an as yet unidentified intracellular mechanism for controlling IFN-I production in neonatal AMs exposed to RSV. However, further investigations are required to define the antiviral mechanism induced by RSV replication and regulated by IRAP in neonates. Neverthless, our study provides a novel insight into RSV-AMs interaction and suggest that targeting IRAP could be a way to improve the IFN-I response to RSV and RSV vaccines in neonates.

## Material and methods

### Virus

As previously described,^30^ recombinant RSV viruses, rRSV-mCherry (RSV-mCherry), and rRSV-Luc (RSV-Luc) corresponding to RSV Long strain expressing either the mCherry or the Luciferase proteins respectively, or recombinant WT RSV Long strain (RSV), were produced on HEp-2 cells. The virus stocks were aliquoted and stored at −80 °C. The infectivity of virus stocks was determined on HEp-2 cell monolayers by standard plaque assay. UV-inactivated-RSV consisted of RSV-mCherry exposed 20 min to ultra-violet (UV). Mock control consisted of HEp-2 cell culture supernatant.

### Animals and viral infection

This study was carried out in accordance with the recommendations of COMETHEA ethics committee. The protocol was approved under authorization number 15–16 (2015060414241349 v1, APAFIS#600). IRAP^KO^ mice and C57BL6/J co-housed control animals were bred and housed under SPF conditions in our animal facilities (IERP, INRA, Jouy-en-Josas). Neonatal (≤6 days) or adult (7–8 weeks) mice received respectively 10 μL or 50 μL of recombinant RSV expressing luciferase (RSV-Luc, 2.34 × 10^6^ pfu/mL)^30^ or mock-infection control by intranasal instillation. Mice were then sacrificed at different timepoints by intraperitoneal (I.P.) injection of pentobarbital and lungs and NT were frozen.

### Bioluminescence measurements

Photon emission was measured in RSV-infected neonatal C57BL/6 mice or in the lungs after extraction using the IVIS system (Xenogen Biosciences) and Living Image software (version 4.0, Caliper Life Sciences). Briefly, neonates received I.P. injection of 50 μL of D-luciferin (30 mg/mL, Perking Elmer) and luciferase activity was measured for 1 min with f/stop = 1 and binning = 8.

### Luciferase expression of lung or nasal turbinate lysates

Frozen lungs or NT were weighed and then homogenized in 300 μL of Passive Lysis Buffer (PLB) (1 mM Tris pH 7.9; 1 mM MgCl2; 1% Triton × 100; 2% glycerol; 1 mM DTT) with a Precellys 24 bead grinder homogenizer (Bertin Technologies, St Quentin en Yvelines, France) and a cycle of 2 × 15 s at 4 m/s, as previously described ^25^ (L. Lung or NT homogenates were clarified by centrifugation 5 min at 2000 × *g* and distributed on microplates (100 µL). Then, 100 µL of D-luciferine (1 μg/mL, Sigma-Aldrich) in PLB complemented with 0.5 mM of ATP were added on each well. Photon emission was measured using an In Vivo Imaging System (IVIS-200, Xenogen, Advanced Molecular Vision) and Live Imaging software (version 4.0, Caliper Life Sciences). Data were expressed in radiance (photons/sec/cm^2^/sr) and normalized to the weight of homogenized tissues.

### AMs isolation, RSV exposure, and TLR/RLR stimulation

A cannula was inserted in trachea from adult or neonatal mice and repeated BAL were made with PBS. AMs were isolated after centrifugations (≈2 × 10^4^ AMs in BAL from 1 neonate *versu*s ≈1 × 10^5^ AMs in BAL from 1 adult mouse) and 1 × 10^5^ AMs were plated in 96-well cell culture plates in RPMI supplemented with L-glutamine 2 mM, FCS 5%, and antibiotics for 24 h to allow for adhesion. AMs were then exposed to recombinant RSV expressing mCherry^30^ or not (WT rRSV) or UV-inactivated mCherry-RSV (the same batch exposed 20 min to UV) at multiplicity of infection (MOI) = 5 or Hep2 cell culture supernatant (Mock), or stimulated with imiquimod 100 µg/mL (Invivogen), CpG-B 100 µg/mL (Sigma-Aldrich), Poly(I:C) LMW 100 µg/mL (Invivogen), LPS 10 µg/mL (E. coli 0111:B4, Sigma-Aldrich) or Poly(I:C)-LMW/LyoVec 0.1 or 1 µg/mL (Invivogen). In some experiment, adult C57BL/6 AMs were infected in the presence of anti-IFNAR1 antibody (BioXcell, MAR1-5A3, 5 or 10 µg/mL) or control isotype (MOPC-21). After 24 h, supernatants were collected and cells were frozen in NucleoSpin^®^RNA XS Kit (Macherey-Nagel) lysis buffer for qRT-PCR analysis. Cell preparations from WT or deficient mice (pups or adults) were performed simultaneously. However, the analyzes were separated for a better understanding of results.

#### Flow cytometry

After BAL or macrophages culture, cells were stained in FACS buffer (PBS, SVF 3%, 2 mM EDTA) with Fc receptor-Block (SONY Biotechnolgy, clone 93, cat# 1106600, dilution 1:100) and extracellular markers for 20 min in dark at 4 °C: PE-conjugated anti-mouse CD86 (BD Biosciences, clone GL1, cat# 561963, dilution 1:100), PE-Cy7-conjugated anti-mouse CD11c (SONY, clone N418, cat# 1186585, dilution 1/600), PerCP-Cy5.5-conjugated anti-mouse EpCAM (SONY, clone G8.8, cat# 1191095, dilution 1/100), Alexa fluor 647-conjugated anti-mouse CD11b (BD Biosciences, clone M1/70, cat# 557686, dilution 1:100), APC-Cy7-conjugated anti-mouse CD45.2 (Biolegend, clone 104, cat# 109823, dilution 1:100), and BV421-conjugated anti-mouse Siglec-F (BD Biosciences, clone E50-2440, cat# 562681, dilution 1:100). Cells were then washed with FACS buffer and centrifugated at 1800 rpm 10 min at 4 °C. Cells were suspend in PBS and immediately acquired using a LSR Fortessa with DIVA software (BD Biosciences). Data were analyses using FlowJO v.10 software (Treestar).

### Viral N-RNA load and gene expression by qRT-PCR

Frozen lungs were homogenized in NucleoSpin^®^RNA XS Kit (Macherey-Nagel) lysis buffer with a Precellys 24 bead grinder homogenizer (Bertin Technologies, St Quentin en Yvelines, France). Total RNA was extracted from lung and cell lysates using NucleoSpin^®^ RNA (XS) kit (Macherey-Nagel) and reverse transcribed using the iScript™ Reverse Transcription Supermix for RT-qPCR kit (Bio-Rad) according to the manufacturer’s instructions. The primers (Sigma-Aldrich) used are listed in Table 1. For in vivo expriments, *GADPH* and *HPRT* were used as house-keeping gene. The qPCRs were performed with the MasterCycler RealPlex (Eppendorf) and SYBRGreen PCR Master Mix (Eurogenetec) and data analyzed with the Realplex software (Eppendorf) to determine the cycle threshold (Ct) values. Results were determined with the formula 2^−ΔCt^ with ΔCT = Ct_gene_ − Ct_GADPH_ or are expressed in fold-change calculated by the formula 2^−ΔΔCt^ with ΔΔCT = ΔCT_RSV_/ΔCT_mock_. ΔCT_mock_ represented the mean of control conditions.

**Table 1:** List of primers.

Name of gene	Forward primer (5′ to 3′)	Reverse primer (5′ to 3′)
*GAPDH*	GGGGTCGTTGATGGCAACA	AGGTCGGTGTGAACGGATTTG
*bActine*	TGT TAC CAA CTG GGA CGA CA	GGG GTG TTG AAG GTC TCA AA
*N (huRSV-A2)*	AGATCAACTTCTGTCATCCAGCAA	TTCTGCACATCATAATTAGGAGTATCAAT
*OAS 1a*	CTTTGATGTCCTGGGTCATGT	GCTCCGTGAAGCAGGTAGAG
*ISG15*	CCCCAGCATCTTCACCTTTA	TGACTGTGAGAGCAAGCAGC
*IFITM3*	TTCAGGCACTTAGCAGTGGA	AGCCTATGCCTACTCCGTGA
*IRF7*	CTGGATGAAGAAGATGCACAG	GAAGTTGGTCTTCCAGCCTC
*RIG-I*	TGGCTTCACAAAGTCCACAG	CTGCCTCACTCTTCCTCCAG
*TLR3*	ATAGGGACAAAAGTCCCCCA	ATGATACAGGGATTGCACCC
*TLR4*	TGTCATCAGGGACTTTGCTG	GGACTCTGATCATGGCACTG
*TLR7*	CCACAGGCTCACCCATACTTC	GGGATGTCCTAGGTGGTGACA
*TLR9*	CTGTACCAGGAGGGACAAGG	CAGTTTGTCAGAGGGAGCCT

### Cytokine quantification

IFN-α and IFN-β were measured in supernatants of AMs or lung lysates using IFN alpha/IFN beta 2-Plex Mouse ProcartaPlex™ immunoassay (ebiosciences). Data were acquired using a Bio-Plex^®^ multiplex system (Bio-Rad) in order to determine the mean of fluorescent intensities (MFIs) and results were analyzed on ProcartaPlex^®^ Analyst software. For in vivo experiments, concentrations were normalized to weight lungs. IL-6 was quantified by ELISA (Ready-SET-Go, eBiosciences) according to the manufacturer’s instructions.

### Immunofluorescence microscopy

AMs were seeded on fibronectin-coated slides and stained as previously described.^23^ Cells were fixed with 4% PFA and permeabilized with 0.2% saponin in PBS containing 0.2% BSA and stained in the same buffer. Cells were stained with rabbit monoclonal anti-IRAP (clone D7C5, Cell signaling), anti-early endosome antigen 1 (EEA1, N-19, clone sc-6415, Santa Cruz), alexa 488 coupled-donkey anti-mouse antibody, donkey anti-goat Alexa 594 (A32758, Molecular Probes), and DAPI. Images were acquired on a Leica SP8 confocal microscope. Image treatment and analysis were performed with ImageJ software.

### Statistical analysis

Nonparametric Mann–Whitney test (comparison of two groups, *n* ≥ 4) was used to compare unpaired values (GraphPad Prism software, version 8.4.3). Significance is represented: *p < 0.05; **p < 0.01; ***p < 0.001; ****p < 0.0001.

## Supplementary Information

Supplementary Figures

## References

[1] Smyth RL, Openshaw PJ (2006). Bronchiolitis. Lancet.

[2] Janssen R (2007). Genetic susceptibility to respiratory syncytial virus bronchiolitis is predominantly associated with innate immune genes. J. Infect. Dis..

[3] Lambert L, Sagfors AM, Openshaw PJ, Culley FJ (2014). Immunity to RSV in early-life. Front Immunol..

[4] Cormier SA, You D, Honnegowda S (2010). The use of a neonatal mouse model to study respiratory syncytial virus infections. Expert Rev. Anti Infect. Ther..

[5] de Kleer IM (2016). Perinatal activation of the interleukin-33 pathway promotes Type 2 immunity in the developing lung. Immunity.

[6] Saluzzo S (2017). First-breath-induced Type 2 pathways shape the lung immune environment. Cell Rep..

[7] Drajac C, Laubreton D, Riffault S, Descamps D (2017). Pulmonary susceptibility of neonates to respiratory syncytial virus infection: a problem of innate immunity?. J. Immunol. Res..

[8] Culley FJ, Pollott J, Openshaw PJ (2002). Age at first viral infection determines the pattern of T cell-mediated disease during reinfection in adulthood. J. Exp. Med..

[9] Remot A (2016). Flt3 ligand improves the innate response to respiratory syncytial virus and limits lung disease upon RSV reexposure in neonate mice. Eur. J. Immunol..

[10] Ruckwardt TJ, Malloy AM, Morabito KM, Graham BS (2014). Quantitative and qualitative deficits in neonatal lung-migratory dendritic cells impact the generation of the CD8^+^ T cell response. PLoS Pathog..

[11] Stephens, L. M. & Varga, S. M. Function and modulation of type I interferons during respiratory syncytial virus infection. *Vaccines* **8** (2020). 10.3390/vaccines8020177 PMC7349809 32290326

[12] Hall CB, Douglas RG, Simons RL, Geiman JM (1978). Interferon production in children with respiratory syncytial, influenza, and parainfluenza virus infections. J. Pediatr..

[13] Cormier SA (2014). Limited type I interferons and plasmacytoid dendritic cells during neonatal respiratory syncytial virus infection permit immunopathogenesis upon reinfection. J. Virol..

[14] Hijano DR (2019). Role of Type I Interferon (IFN) in the respiratory syncytial virus (RSV) immune response and disease severity. Front Immunol..

[15] Makris S, Paulsen M, Johansson C, Type I (2017). Interferons as regulators of lung inflammation. Front Immunol..

[16] Goritzka M (2014). Alpha/beta interferon receptor signaling amplifies early proinflammatory cytokine production in the lung during respiratory syncytial virus infection. J. Virol..

[17] Zeng R, Cui Y, Hai Y, Liu Y (2012). Pattern recognition receptors for respiratory syncytial virus infection and design of vaccines. Virus Res..

[18] Miyake K (2018). Mechanisms controlling nucleic acid-sensing Toll-like receptors. Int. Immunol..

[19] Saitoh SI (2017). TLR7 mediated viral recognition results in focal type I interferon secretion by dendritic cells. Nat. Commun..

[20] Liu Y, Olagnier D, Lin R (2016). Host and viral modulation of RIG-I-mediated antiviral immunity. Front Immunol..

[21] Ohman T, Rintahaka J, Kalkkinen N, Matikainen S, Nyman TA (2009). Actin and RIG-I/MAVS signaling components translocate to mitochondria upon influenza A virus infection of human primary macrophages. J. Immunol..

[22] Saveanu L (2009). IRAP identifies an endosomal compartment required for MHC class I cross-presentation. Science.

[23] Babdor J (2017). IRAP+ endosomes restrict TLR9 activation and signaling. Nat. Immunol..

[24] Goritzka M (2015). Alveolar macrophage-derived type I interferons orchestrate innate immunity to RSV through recruitment of antiviral monocytes. J. Exp. Med..

[25] Laubreton, D. et al. Regulatory B lymphocytes colonize the respiratory tract of neonatal mice and modulate immune responses of alveolar macrophages to RSV Infection in IL-10-dependant manner. *Viruses* **12**, 822 (2020). 10.3390/v12080822 PMC7472339 32751234

[26] Nikolaou A (2014). Presence and regulation of insulin-regulated aminopeptidase in mouse macrophages. J. Renin Angiotensin Aldosterone Syst..

[27] Makris S, Bajorek M, Culley FJ, Goritzka M, Johansson C (2016). Alveolar macrophages can control respiratory syncytial virus infection in the absence of Type I interferons. J. Innate Immun..

[28] Prince GA, Horswood RL, Berndt J, Suffin SC, Chanock RM (1979). Respiratory syncytial virus infection in inbred mice. Infect. Immun..

[29] Chavez-Bueno S (2005). Respiratory syncytial virus-induced acute and chronic airway disease is independent of genetic background: an experimental murine model. Virol. J..

[30] Rameix-Welti MA (2014). Visualizing the replication of respiratory syncytial virus in cells and in living mice. Nat. Commun..

[31] Ravi LI (2013). A systems-based approach to analyse the host response in murine lung macrophages challenged with respiratory syncytial virus. BMC Genom..

[32] Schneider WM, Chevillotte MD, Rice CM (2014). Interferon-stimulated genes: a complex web of host defenses. Annu Rev. Immunol..

[33] Howes A (2016). Differential production of Type I IFN determines the reciprocal levels of IL-10 and proinflammatory cytokines produced by C57BL/6 and BALB/c macrophages. J. Immunol..

[34] Kenney AD (2017). Human genetic determinants of viral diseases. Annu Rev. Genet.

[35] Malinczak, C. A., Lukacs, N. W. & Fonseca, W. Early-life respiratory syncytial virus infection, trained immunity and subsequent pulmonary diseases. *Viruses* **12** (2020). 10.3390/v12050505 PMC7290378 32375305

[36] Aksoy E (2007). Interferon regulatory factor 3-dependent responses to lipopolysaccharide are selectively blunted in cord blood cells. Blood.

[37] Danis B (2008). Interferon regulatory factor 7-mediated responses are defective in cord blood plasmacytoid dendritic cells. Eur. J. Immunol..

[38] Collins PL, Melero JA (2011). Progress in understanding and controlling respiratory syncytial virus: still crazy after all these years. Virus Res..

[39] Sedeyn K, Schepens B, Saelens X (2019). Respiratory syncytial virus nonstructural proteins 1 and 2: exceptional disrupters of innate immune responses. PLoS Pathog..

[40] Hillyer P (2017). Respiratory syncytial virus infection induces a subset of types I and III interferons in human dendritic cells. Virology.

[41] Ling Z, Tran KC, Teng MN (2009). Human respiratory syncytial virus nonstructural protein NS2 antagonizes the activation of beta interferon transcription by interacting with RIG-I. J. Virol..

[42] Spann KM, Tran KC, Collins PL (2005). Effects of nonstructural proteins NS1 and NS2 of human respiratory syncytial virus on interferon regulatory factor 3, NF-kappaB, and proinflammatory cytokines. J. Virol..

[43] Spann KM, Tran KC, Chi B, Rabin RL, Collins PL (2004). Suppression of the induction of alpha, beta, and lambda interferons by the NS1 and NS2 proteins of human respiratory syncytial virus in human epithelial cells and macrophages [corrected]. J. Virol..

[44] Quicke KM, Diamond MS, Suthar MS (2017). Negative regulators of the RIG-I-like receptor signaling pathway. Eur. J. Immunol..

[45] Si-Tahar M (2014). Protective role of LGP2 in influenza virus pathogenesis. J. Infect. Dis..

[46] Evnouchidou I (2020). IRAP-dependent endosomal T cell receptor signalling is essential for T cell responses. Nat. Commun..

[47] Weimershaus M (2012). Conventional dendritic cells require IRAP-Rab14 endosomes for efficient cross-presentation. J. Immunol..

[48] Liegeois M, Legrand C, Desmet CJ, Marichal T, Bureau F (2018). The interstitial macrophage: A long-neglected piece in the puzzle of lung immunity. Cell Immunol..

[49] Rincheval V (2017). Functional organization of cytoplasmic inclusion bodies in cells infected by respiratory syncytial virus. Nat. Commun..

[50] Morris S (2011). Autophagy-mediated dendritic cell activation is essential for innate cytokine production and APC function with respiratory syncytial virus responses. J. Immunol..

[51] Reed M (2013). Autophagy-inducing protein beclin-1 in dendritic cells regulates CD4 T cell responses and disease severity during respiratory syncytial virus infection. J. Immunol..

[52] Reed M, Morris SH, Owczarczyk AB, Lukacs NW (2015). Deficiency of autophagy protein Map1-LC3b mediates IL-17-dependent lung pathology during respiratory viral infection via ER stress-associated IL-1. Mucosal Immunol..

[53] Pokharel SM, Shil NK, Bose S (2016). Autophagy, TGF-beta, and SMAD-2/3 signaling regulates interferon-beta response in respiratory syncytial virus infected macrophages. Front. Cell. Infect. Microbiol..

[54] Kuma A (2004). The role of autophagy during the early neonatal starvation period. Nature.

[55] Stranks AJ (2015). Autophagy controls acquisition of aging features in macrophages. J. Innate Immun..

[56] Hirata Y (2011). Vimentin binds IRAP and is involved in GLUT4 vesicle trafficking. Biochem. Biophys. Res. Commun..

[57] Tojo H (2003). The Formin family protein, formin homolog overexpressed in spleen, interacts with the insulin-responsive aminopeptidase and profilin IIa. Mol. Endocrinol..

[58] Brock SC, Goldenring JR, Crowe JE (2003). Apical recycling systems regulate directional budding of respiratory syncytial virus from polarized epithelial cells. Proc. Natl Acad. Sci. U.S.A..

[59] Larance M (2005). Characterization of the role of the Rab GTPase-activating protein AS160 in insulin-regulated GLUT4 trafficking. J. Biol. Chem..

[60] Szatmari Z, Sass M (2014). The autophagic roles of Rab small GTPases and their upstream regulators: a review. Autophagy.

[61] Utley TJ (2008). Respiratory syncytial virus uses a Vps4-independent budding mechanism controlled by Rab11-FIP2. Proc. Natl Acad. Sci. U.S.A..

