# Supplementary figures and images for: Control of IFN-I responses by the aminopeptidase IRAP in neonatal C57BL/6 alveolar macrophages during RSV infection
Source: Mucosal Immunol. 2021 Apr 12;14(4):949–62. doi: 10.1038/s41385-021-00402-w (PMC8221999; doi:10.1038/s41385-021-00402-w)

Supplementary Figure 1

Bioluminescence activity

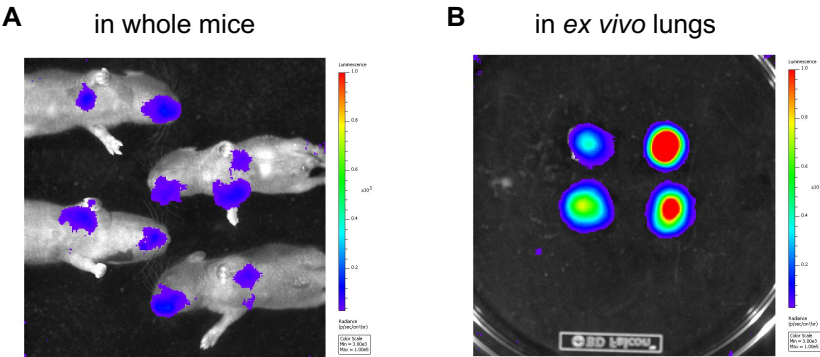

# Supplemental Figure 2

**A**

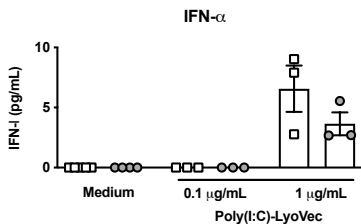

**B**

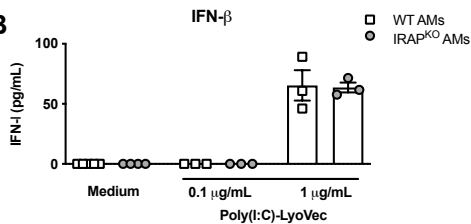

**C**

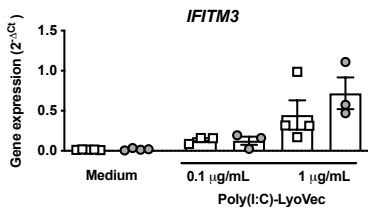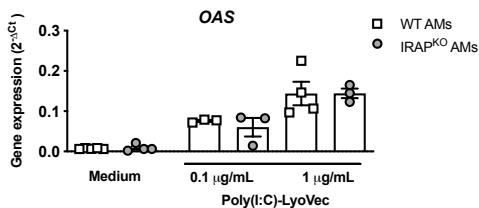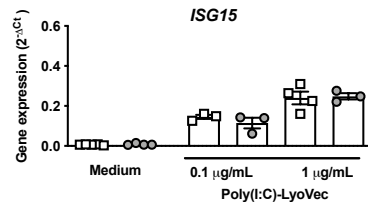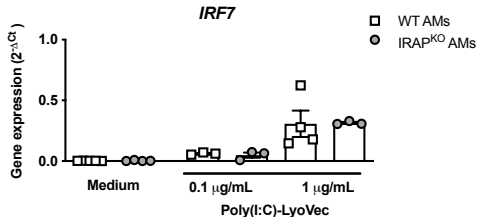

Supplement: Supplementary file 1 — Supplementary Figures [file 41385_2021_402_MOESM1_ESM.pdf]
